# *Cistanche tubulosa* glycosides ameliorate cognitive decline in APP/PS1 mice via modulation of gut microbiota and fatty acid metabolism: insights from multi-omics and experimental validation

**DOI:** 10.3389/fphar.2025.1662336

**Published:** 2025-08-08

**Authors:** Rui Hou, Wei Song, Yi Nan, Yiyi Gong, Jieying Liu, Jialin Liu

**Affiliations:** ^1^ Department of General Surgery, Peking Union Medical College Hospital, Chinese Academy of Medical Sciences and Peking Union Medical College, Beijing, China; ^2^ Center for Biomarker Discovery and Validation, National Infrastructures for Translational Medicine, Institute of Clinical Medicine, Peking Union Medical College Hospital, Chinese Academy of Medical Science and Peking Union Medical College, Beijing, China; ^3^ Graduate School, Tianjin University of Traditional Chinese Medicine, Tianjin, China

**Keywords:** *Cistanche tubulosa*, Alzheimer’s disease, phenylethanoid glycosides, gut microbiota, fatty acids, *Akkermansia*

## Abstract

**Objective:**

The dried succulent stem of *C. tubulosa* (Schenk) Wight has long been used as herbal medicine in China and other regions of Asia for its tonifying properties. This study aimed to elucidate the pharmacological mechanisms of the total glycosides from *Cistanche tubulosa* (GCT) in ameliorating cognitive decline, with a focus on gut microbiota remodeling and metabolic regulation.

**Methods:**

Six-month-old APP/PS1 double-transgenic mice received oral GCT at three doses or donepezil for 60 days. Cognitive function was assessed by the Morris water maze. Aβ burden and inflammatory factors were evaluated by immunohistochemistry and ELISA. Gut microbiota was analyzed using 16S rRNA sequencing. Metabolomic profiles of mice serum and brain were profiled by a targeted metabolomics approach that enabled simultaneous quantitation of 306 metabolites. The effect of GCT on pure-cultured bacterial strain was assessed via growth curve analysis *in vitro*.

**Results:**

GCT treatment significantly improved spatial memory and reduced the protein levels of Aβ and proinflammatory factors in APP/PS1 mice. Multi-omics analyses revealed that GCT rapidly enriched beneficial taxa like *Akkermansia* and suppresses *Firmicutes* since the seventh day of intervention, leading to increased neuroprotective short-chain fatty acids (e.g., β-hydroxybutyrate) and decreased pro-inflammatory long-chain fatty acids in both serum and brain. Crucially, *in-vitro* experiments demonstrated that GCT directly promoted the proliferation of *Akkermansia muciniphila*, a key probiotic implicated in AD amelioration.

**Conclusion:**

This work uncovers a novel “gut microbiota-fatty acid metabolism-neuroinflammation” axis as the primary mechanism underlying GCT’s anti-AD effects. These findings highlight GCT’s therapeutic potential and offer new mechanistic insights into how low-bioavailability phytochemicals exert systemic benefits via the gut-brain axis.

## 1 Introduction

Alzheimer’s disease (AD), a progressive neurodegenerative disorder, poses a significant global health burden due to its irreversible cognitive decline and high prevalence (Abbott, 2023; Scheltens et al., 2021). Over 55 million individuals worldwide currently live with dementia, with AD accounting for 60%–70% of cases, yet existing treatments only offer transient symptomatic relief (Leng and Edison, 2021). Recent research has highlighted the gut-brain axis as a pivotal mediator in AD pathogenesis, with dysbiosis of the gut microbiota emerging as a potential therapeutic target (Jamerlan et al., 2025; Zhang et al., 2023). *Akkermansia muciniphila*, an abundant gut inhabitant of humans and many other animals, have been proven to alleviate dementia-like symptoms in AD mice (Ou et al., 2020; Wang et al., 2025). Gut microbiota-derived metabolites, such as short-chain fatty acids (SCFAs), amino acids, and secondary bile acids, can traverse the intestinal barrier and influence neuroinflammation, amyloid-β (Aβ) aggregation, and tau hyperphosphorylation via circulatory, neural, and immune pathways (Ameen et al., 2022; Chen et al., 2022). Notably, systemic and cerebral metabolic disturbances, including dysregulated lipid, SCFAs, and energy metabolism, have been consistently observed in patients with AD, further implicating gut microbiome-host metabolic crosstalk in disease progression (Butterfield and Halliwell, 2019; Zhang et al., 2024).

Traditional herbal medicines have emerged as promising therapeutic strategy employed in treating mild to moderate dementia as well as in managing AD (Li et al., 2022; Long et al., 2024; Maisto and Mango, 2024). Cistanches Herba (Roucongrong in Chinese), a widely utilized traditional Chinese medicine (TCM), is derived from the dried succulent stems of *Cistanche deserticola* (Y. C. Ma) and *Cistanche tubulosa* (Schenk) Wight, both officially authenticated in the Pharmacopoeia of the People’s Republic of China (The Commission of Chinese Pharmacopoeia, 2020). As described in the Pharmacopoeia, Cistanches Herba is effective in tonifying brain and kidney yang, benefiting essence and blood, and lubricating the intestines. It has long been used for anti-aging and treating forgetfulness in the clinical practice of TCM since the 15th century (Fang et al., 2017; Wang et al., 2017; Song et al., 2021). Glycosides, mainly phenylethanoid glycosides (PhGs) and iridoid glycosides, have been recognized as the predominant constituents in Cistanches Herba (Jiang and Tu, 2009; Zou et al., 2017; Bai et al., 2022). As further development based on its traditional efficacy, the total glycosides from *C. tubulosa* has been used as *C. tubulosa* glycoside capsule (Memoregain^®^) for AD treatment in clinic (Guo et al., 2013). Although PhGs from *C. tubulosa* have exhibited potent neuroprotective effects on different models of cognitive impairment (Li N. et al, 2015; Zhang et al., 2017) and depressive disorder (Li et al., 2018), these compounds demonstrated quite low oral bioavailability (e.g., echinacoside and verbascoside, <1%) (Li Y. et al, 2015; Wu et al., 2020). The pharmacological mechanism of Cistanches Herba in improving cognition remains further explored, and the low oral bioavailability of these glycosides poses a pharmacokinetic paradox.

Intriguingly, emerging evidence suggests that modulation of gut microbiota and microbial metabolites may be a fundamental mechanism through which herbal medicines exert systemic health benefits (Hui et al., 2024; Su et al., 2023; Bai et al., 2024). GCT (with PhGs as major components) has been shown to modulate the composition and metabolism of gut microbiota in rodent models of diabetic nephropathy (Ma et al., 2025), alcoholic liver disease (Qi et al., 2025), and liver fibrosis (Qi et al., 2024). Recent studies also indicated that both TCM formulas and single medicinal herbs can improve cognitive function through the gut-brain axis (Chen et al., 2023; Gou et al., 2023; Wu et al., 2022; Zhao et al., 2025). This paradigm shift raises the possibility that *Cistanche*-derived glycosides may similarly act through remodeling gut microbiome and regulation metabolism to attenuate cognitive decline and AD pathology, which was closely related to the traditional use of Cistanches Herba as “nourishing the brain and enhancing intelligence” in TCM.

In this study, we investigated the effects of *C. tubulosa* glycosides on cognitive function and AD pathology in APPswe/PS1dE9 double-transgenic (APP/PS1) mice, a well-established model characterized by Aβ deposition and cognitive deficits. By integrating 16S rRNA sequencing, targeted metabolomic profiling, and experimental validation *in vivo* and *in vitro*, we elucidated the shifts in gut microbiota and systemic metabolic reprogramming induced by GCT intervention, revealing the mechanism by which *C. tubulosa* glycosides alleviate cognitive decline and Aβ burden in AD mice.

## 2 Materials and methods

### 2.1 Chemicals and reagents

The reference standards of dulcitol, 8-epiloganic acid, and 8-epi-loganic acid-6′-O-*β*-D-glucoside were purchased form Dexter biotechnology Co., Ltd. (Chengdu, China); echinacoside, acteoside, tubuloside A, isoacteoside, 2′-acetylacteoside were purchased form Purifa Technology Development Co., Ltd. (Chengdu, China). Their purities were no less than 95%. The stock solutions of the eight standards were mixed to prepare a standard mixture. Formic acid, methanol, and acetonitrile (ACN) were of LC–MS grade and from Thermo-Fisher (Pittsburgh, USA). Ultrapure water was prepared through Milli-Q water purification system (Millipore, MA). Other reagents and chemicals were of analytical grade.

### 2.2 Preparation of GCT

Raw drug materials, the dried succulent stems of *C. tubulosa*, were collected from Tongrentang Co., Ltd. (Beijing, China), and identified by Dr. Wei Song. The plant voucher is deposited in the national infrastructures for translational medicine (Peking union medicine college hospital). Briefly, the drug materials were sliced, powdered, and then refluxed with 10-fold 75% ethanol for three times (1 h each time) followed by filtration. The filtrates were combined before subjected to frozen drying to obtain GCT extract. For ultra-high performance liquid chromatography coupled with quadruple time-of-flight mass spectrometry (UPLC–qTOF-MS) analysis, an aliquot of 0.1 mg extract was diluted to 10 mL with 75% methanol and then filtered through a 0.22 μm membrane.

### 2.3 UPLC-qTOF-MS analysis of GCT

UPLC-qTOF-MS analysis was performed on an Acquity UPLC™ system coupled with a Synapt G1 MS system (Waters, Milford, MA, USA). A Waters Acquity UPLC HSS T_3_ column (100 × 2.1 mm, 1.8 μm) was used for the analysis, with the column temperature was set at 30 °C. Mobile phases were water with 0.1% formic acid (A) and acetonitrile (B). The gradient used was as follow: (0–5.0) min, 5%–10% B; 5.0–6.0 min, 10%–13% B; 6.0–12.0 min,13%–15% B; 12.0–14.0 min, 15%–17% B; 14.0–17.0 min, 17%–23% B; 17.0–21.0 min, 23%–35% B; 21.0–22.0 min, 35%–95% B; 22.0–24.0 min, 95% B. The flow rate was 0.5 mL/min. The injection volum of sample was 1 μL. The data acquisiton mode was MS^E^. The analysis Data was acquired from 50 to 1,500 Da. The source temperature was 100°C, and the desolvation temperature was 450°C, with desolvation gas flow 850 L/h, Leucine enkephaline was used as lock mass, The capillary voltage was 3 kV. At low CE scan, the cone voltage was 30 V for (−) ESI. The collision energy was 6 eV (trap) and 4 eV (transfer), and the collision energy was 20˗45 eV ramp (trap) and 12 eV (transfer) for (−) ESI. The instrument was controlled by Masslynx 4.1 software (Waters, Milford, MA, USA).

### 2.4 Mice grouping and treatment

Six-month-old male APP/PS1 mice and C57BL/6J wild type (WT) controls were obtained from Huafukang Biotechnology Co., Ltd. (Beijing, China). Animals were maintained under specific pathogen-free conditions with controlled temperature (22°C ± 1°C) and a 12-h light/dark cycle. After 1 week of acclimatization, APP/PS1 mice were randomly allocated into five groups (n = 10 each group): APP/PS1 model, APP/PS1 + low-dose GCT (CTL, 100 mg kg^-1^·d^-1^), APP/PS1 + medium-dose GCT (CTM, 200 mg kg^-1^·d^-1^), APP/PS1 + high-dose GCT (CTH, 400 mg kg^-1^·d^-1^), and APP/PS1 + positive drug donepezil (0.92 mg kg^-1^·d^-1^). Age-matched WT mice (n = 10) served as normal controls. GCT powder and donepezil were freshly suspended in distilled water (1.0 mL) for daily oral gavage administration. Both the untreated APP/PS1 model group and WT control group received equivalent volumes of distilled water. Six out of the 50 APP/PS1 mice (one in model group, two in CTL group, one in CTM group, one in CTH group, and one in donepezil group) unexpectedly died, while none of the WT mice died within the treatment period continued for 60 consecutive days.

### 2.5 Morris water maze (MWM) testing

Spatial learning and memory were assessed using the Morris water maze (MWM) according to established protocols (Morris R, 1984). The experiment commenced on the 55th day post-treatment and consisted of a 5-day training stage followed by a probe trial on day 6. The apparatus comprised a circular pool (120 cm diameter) filled with water maintained at 22°C ± 1°C. Edible white pigment was added to water to track the mice accurately. A removable escape platform (10 cm diameter) was positioned 1 cm below the water surface in the center of a fixed target quadrant. During the acquisition phase, mice underwent four trials per day. Each trial began by placing the mouse facing the pool wall at one of four randomized entry points (northeast, northwest, southeast, southwest quadrant). Animals were allowed 60 s to locate the platform, after which they were gently guided to it and remained for 10 s if unsuccessful. Escape latency (time to reach the platform) and swim speed were recorded when the mice found and resided on the platform. On day 6, all mice were subjected to the probe trial in which they swam for 60 s in the pool without the platform. Spatial memory retention was quantified by measuring time spent in the target quadrant and number of crossings through the former platform location using an automated video tracking system.

### 2.6 Sample collection

The fecal samples were gathered at the 7th, 30th, and 60th day of treatment to analyze the intestinal microbiota and metabolites. After behavioral tests, five mice from each group were sacrificed for sample collection. The serum and cerebral tissues were collected and stored at −80°C until used. The serum was used for biochemical tests and metabolomics analysis. Parts of the brain was used for histomorphometry and the rest was used for metabolomics analysis.

### 2.7 Histopathological assessment

The cerebral tissues were fixed by tissue fixative, washed, dehydrated, and then embedded with paraffin. After sliced into sections (5 μm in thickness), hematoxylin and eosin (H&E) staining and Congo red staining were performed following standard protocols.

### 2.8 Biochemical assay by ELISA

The quantification of soluble Aβ (Aβ40) and insoluble Aβ (and Aβ42) in mice brain was performed using commercial ELISA kits (Invitrogen, KMB3481 for mouse Aβ40 and KMB3441 for mouse Aβ42, respectively). The serum and cerebral levels of interleukin-1β (IL-1β), interleukin-6 (IL-6), and tumor necrosis factor-α (TNF-α) were determined using IL-1β ELISA Kit (Halic, DRE30027), IL-6 ELISA Kit (Halic, DRE30044), and TNF-α ELISA Kit (Halic, DRE30030) for mouse, respectively. The kits were utilized as per manufacturer’s instructions.

### 2.9 16S rRNA gene microbiome sequencing analysis

The total microbial genomic DNA was extracted from mice fecal samples collected at the 7th day and 60th day using the FastPure stool DNA isolation Kit (MJYH, shanghai, China) according to manufacturer’s instructions. The quality and concentration of DNA were determined by 1.0% agarose gel electrophoresis and a NanoDrop2000 spectrophotometer (Thermo Scientific, United States). The V3-V4 hypervariable region of the 16S rRNA gene were amplified with primer pairs 338F (5′-ACT​CCT​ACG​GGA​GGC​AGC​AG-3′) and 806R (5′-GGACTACHVGGGTWTCTAAT-3′). The 250-bp amplicons were sequenced using the Illumina Nextseq2000 platform (Illumina, San Diego, USA) according to the standard protocols by Majorbio Bio-Pharm Technology Co. Ltd. (Shanghai, China). Raw FASTQ files were de-multiplexed and then quality-filtered by fastp (https://github.com/OpenGene/fastp) and merged by FLASH (http://www.cbcb.umd.edu/software/flash). Then the optimized sequences were clustered into operational taxonomic units (OTUs) using UPARSE (http://drive5.com/uparse/) and analyzed using Majorbio Cloud platform (https://cloud.majorbio.com).

### 2.10 Targeted metabolomics analysis

The targeted metabolomics analysis of mice serum and cerebral samples was conducted with the Q300 Metabolite Array Kit (Metabo-Profile Biotechnology, Shanghai, China). Briefly, reference standards of targeted metabolites and extractions from samples were prepared according to the instructions of the kit as previously reported (Xie et al., 2021). Methods for sample preparation were summarized in Supplementary Material. An ultra-high performance liquid chromatography coupled with tandem mass spectrometry (UPLC-MS/MS) system (ACQUITY UPLC-Xevo TQ-S, Waters, Milford, MA, USA) was used to quantitate all targeted metabolites. Samples were separated on an ACQUITY UPLC BEH C18 column (2.1 × 100 mm, 1.7 μm, Waters, MA, USA). The mobile phase consisted of water containing 0.1% formic acid (v/v, A) and acetonitrile/IPA (7:3, v/v, B). The following linear elution gradient was used: 0–1 min (5% B), 1–11min (5%–78% B), 11–13.5 min (78%–95% B), 13.5–14 min (95%–100% B), 14–16 min (100% B), 16–16.1 min (100%–5% B), 16.1–18 min (5% B). The flow rate was 0.40 mL/min. The column temperature was maintained at 40°C. The sample tray temperature was maintained at 10°C. For MS detection, the capillary voltage was set at 1500 V in positive ion mode and 2000 V in negative ion mode. The source temperature was set at 550°C, and desolvation gas flow at 1000 L/h. Quantitative analyses of 306 targeted metabolites were monitored in multiple reaction monitoring (MRM) mode. The quality control samples and blank samples were analyzed across the sample set. Raw data files from UPLC-MS/MS were processed using the MassLynx software (v4.1, Waters, Milford, MA, USA) for peak integration, calibration, and metabolite quantitation.

### 2.11 *In-vitro* culture experiment

The *A. muciniphila* (*A.muciniphila*, BNCC341917) was provide by Beina Biotechnology (Beijing, China) and cultured in Brain Heart Infusion (BHI) agar at 37°C in anaerobic environment. The GCT extract was added to BHI medium at different concentrations (0, 3.125, 6.25, 12.5, 25, 50, 100, and 200 μg/mL) to evaluate the effect of GCT on the growth of *A. muciniphila*. The optical density (OD) value (600 nm) of bacterial solution was measured at 0, 4, 8, 12, 16, 24, 36, and 48 h to draw the growth curve.

### 2.12 Statistical analysis and visualization

Statistical analyses were conducted using GraphPad Prism 8.0 (GraphPad, San Diego, CA, USA) or R package. Comparisons of two groups were performed using two-tailed unpaired Student’s t-test. Comparisons of multiple groups were performed using one-way analysis of variance (ANOVA) with Tukey’s test as the posthoc analysis. Data were presented as mean ± SD. Kruskal–Wallis rank sum test was performed to analyze the gut microbiota sequencing data. Differences were considered significant with P values <0.05.

## 3 Results

### 3.1 Chemical characterization of GCT

**FIGURE 1:**
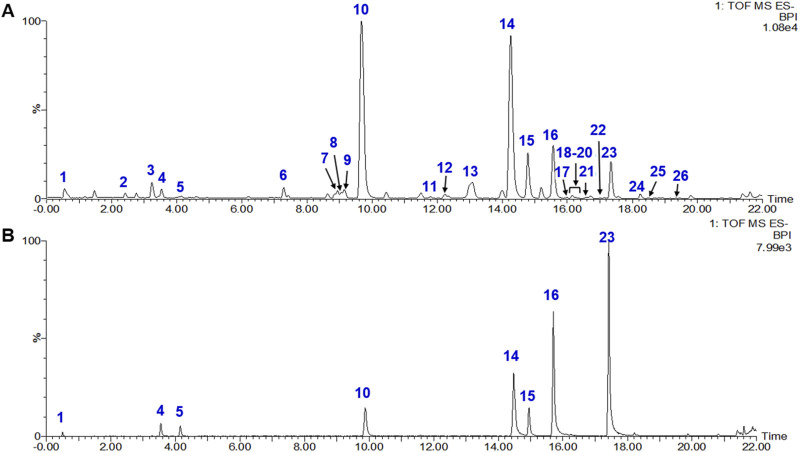
Chemical characterization of the total glycosides extracts from *Cistanche tubulosa*. **(A)** Total ion chromatogram (TIC) of the extract analyzed by UPLC–qTOF-MS. **(B)** TIC of mixture of standards (1, dulcitol; 4, 8-epiloganic acid; 5, 8-Epi-Loganic acid-6′-O-β-D-glucoside; 10, echinacoside; 14, acteoside; 15, tubuloside A; 16, isoacteoside; 23, 2′-acetylacteoside).

A total of 26 constituents in GCT extract were characterized by UPLC-qTOF-MS (Figure 1A, 1˗26). Among them, eight compounds, including dulcitol, 8-epiloganic acid, 8-Epi-loganic acid-6′-O-β-D-glucoside, echinacoside, acteoside, tubuloside A, isoacteoside, and 2′-acetylacteoside (1, 4, 5, 10, 14, 16, and 23 in Figure 1B, respectively), were confirmed by comparing with reference standards. These constituents consisted one monosaccharide (1), two iridoids (2, 3), and 20 PhGs (4˗26), detailed information of which were listed in Table 1. The result above indicated that the GCT extract prepared for further *in-vivo* experiments is abundant with PhGs.

**TABLE 1:** Characterization of chemical constituents in total glycosides extract of *Cistanche tubulosa* by UPLC-Q-TOF/MS analysis.

Peak no.	t_R_ (min)	Identification	Formula	Measured [M-H]^-^ (*m/z*)	Predicted [M-H]^-^ (*m/z*)	Mass error (mDa)	(−) MS^E^ (*m/z*)
1	0.55	Dulcitol	C_6_H_14_O_6_	181.0675	181.0707	3.2	181.0677 [M-H]^-^
2	2.42	geniposide acid	C_16_H_22_O_10_	373.1125	373.1135	−1	211.0651 [M-H-Glu]^-^ 167.0734 [M-H-Glu-CO_2_]^-^ 149.0625 [M-H-Glu-CO_2_-H_2_O]^-^
3	3.24	adoxosidic acid or mussaenoside acid	C_16_H_24_O_10_	375.1261	375.1291	−3	213.0781 [M-H-Glu]^-^ 169.0913 [M-H-Glu-CO_2_]^-^
4	3.54	8-epiloganic acid	C_16_H_24_O_10_	375.1262	375.1291	−2.9	213.0735 [M-H-Glu]^-^ 169.0810 [M-H-Glu-CO_2_]^-^ 151.0739 [M-H-Glu-CO_2_-H_2_O]^-^
5	4.14	cistanoside F	C_21_H_28_O_13_	487.1424	487.1452	−2.8	179.0375 [M-H-Rha-Glu]^-^ 135.0472 [M-H-Rha-Glu-CO_2_]^-^
6	7.29	Cistanbuloside C1/C2	C_35_H_46_O_21_	801.2440	801.2452	−1.2	783.2352[M-H-H_2_O]^-^ 621.1963[M-H-H_2_O-Cf]^-^
7	8.94	campneoside Ⅱ	C_29_H_36_O_16_	639.1952	639.1925	2.7	621.1876 [M-H-H_2_O]^-^ 459.1581[M-H-H_2_O-Cf]^-^
8	9.05	kankanoside K_1_ or kankanoside K_2_	C_36_H_48_O_21_	815.2638	815.261	2.8	783.2398[M-H-OMe]^-^ 621.2006[M-H-OMe-Cf]^-^
9	9.15	isomer of campneoside Ⅱ	C_29_H_36_O_16_	639.1960	639.1925	3.5	621.1908 [M-H-H_2_O]^-^ 459.1581 [M-H-H_2_O-Cf]^-^
10	9.67	echinacoside	C_35_H_46_O_20_	785.2551	785.2504	4.7	623.2177 [M-H-Cf]^-^ 477.1600[M-H-Cf-Rha]^-^
11	11.79	poliumoside or isomer of poliumoside	C_35_H_46_O_19_	769.2538	769.2555	−1.7	607.2241 [M-H-Cf]^-^
12	12.24	poliumoside or isomer of poliumoside	C_35_H_46_O_19_	769.2543	769.2555	−1.2	769.2507 [M-H-Rha]^-^
13	13.08	compneoside Ⅰ	C_30_H_38_O_16_	653.2090	653.2082	0.8	621.1823 [M-H-OMe]^-^ 459.1388 [M-H-OMe-Cf]^-^
14	14.26	acteoside	C_29_H_36_O_15_	623.1982	623.1976	0.6	461.1638[M-H-Cf] 315.1030[M-H-Cf-Rha]^-^
15	14.79	tubuloside A	C_37_H_48_O_21_	827.2728	827.2610	5.8	665.2258[M-H-Cf]^-^ 623.2145[M-H-Ac-Cf]^-^
16	15.56	isoacteoside	C_29_H_36_O_15_	623.1967	623.1976	−0.9	416.1633[M-H-Cf]^-^ 315.1101[M-H-Cf-Rha]^-^
17	15.99	Isomer of acteoside	C_29_H_36_O_15_	623.1963	623.1976	−1.3	461.1638[M-H-Cf]^-^
18	16.16	syringalide A 3′-*α*-*L*-rhamnopyranoside	C_29_H_36_O_14_	607.2058	607.2027	3.1	461.1549 [M-H-Rha]^-^ 445.1814 [M-H-Cf]^-^ 299.1205 [M-H-Cf-Rha]^-^
19	16.25	2′-O-acetylpoliumoside	C_37_H_48_O_20_	811.2687	811.2661	2.6	665.2257[M-H-Rha]^-^,623.2124[M-H-Rha-AC]^-^
20	16.35	isosyringalide 3′-*α*-*L*-rhamnopyranoside	C_29_H_36_O_14_	607.1942	607.2027	−8.5	461.1740 [M-H-Rha]^-^ 315.1086 [M-H-Rha-Cm]^-^ 163.0414[M-H-Rha-Glu-PhA]^-^
21	16.58	kankanoside Ⅰ	C_35_H_46_O_18_	753.2550	753.2603	−5.3	591.265[M-H-Cf]^-^
22	17.00	kankanoside G	C_29_H_36_O_14_	607.2019	607.2027	−0.8	461.1740[M-H-Rha]^-^ 445.1814 [M-H-Cf]^-^ 299.1205 [M-H-Glu-Ac]^-^
23	17.31	2′-acetylacteoside	C_31_H_38_O_16_	665.2067	665.2082	−1.5	623.2177[M-H-Ac]^-^ 477.1600[M-H-Ac-Cf]^-^
24	18.24	tubulaside B	C_3_1H_38_O_16_	665.2162	665.2082	8	503.1887 [M-H-Cf]^-^ 461.1740 [M-H-Cf-Ac]^-^
25	18.49	tubuloside E	C_31_H_38_O_15_	649.2070	649.2132	−6.2	607.2109 [M-H-Ac]^-^ 461.1713 [M-H-Cf-Ac]^-^ 315.1131 [M-H-Cf-Ac-Rha]^-^
26	19.34	osmanthuside B6 or osmanthuside B	C_29_H_36_O_13_	591.2018	591.2078	−6	445.1787 [M-H-Cf]- 163.0414 [M-H-Rha-Glu-Cm]^-^

### 3.2 GCT rescued cognitive impairment of APP/PS1 mice

**FIGURE 2:**
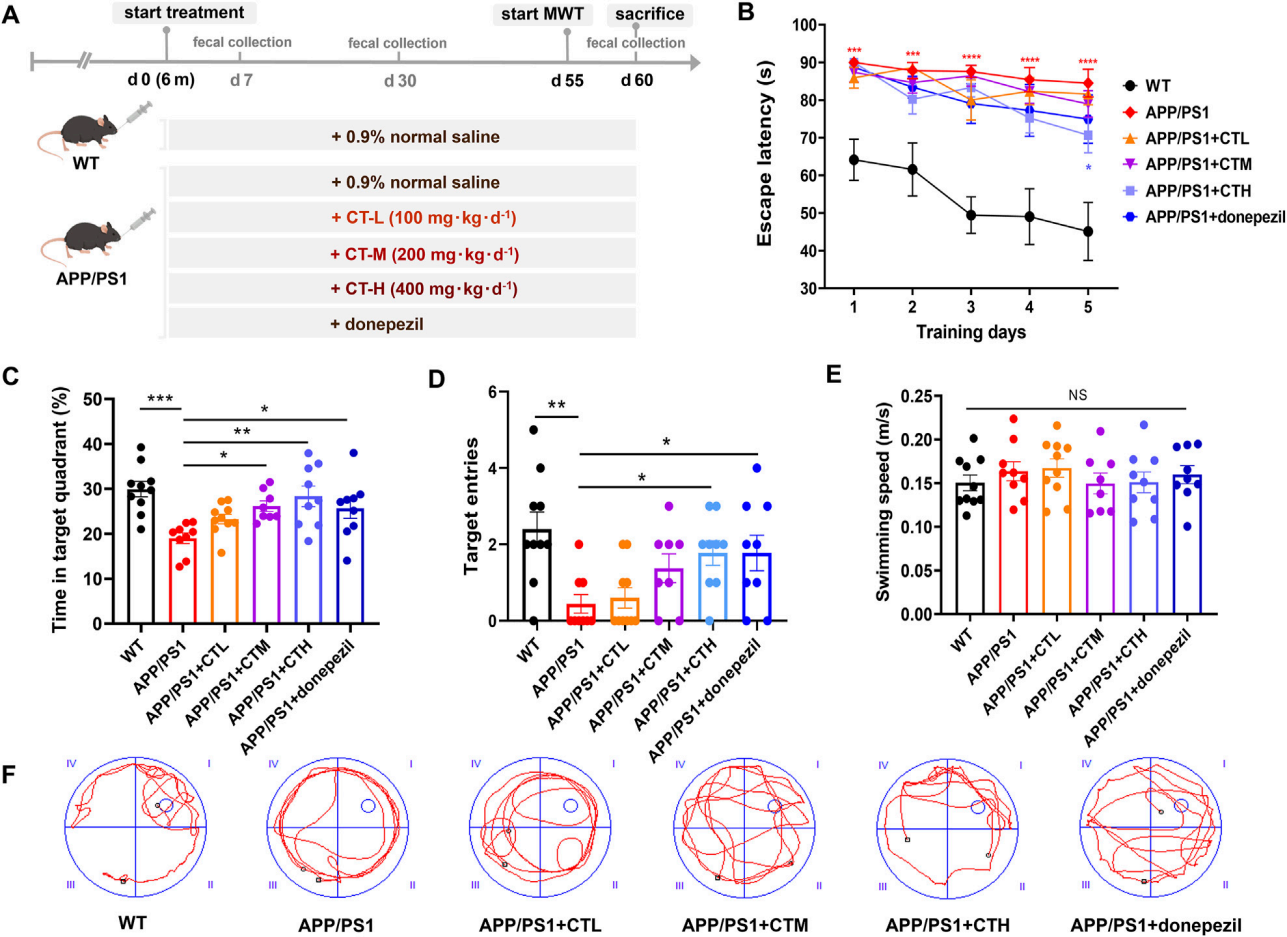
Total glycosides of *Cistanche tubulosa* improved the spatial learning and memory of APP/PS1 mice. **(A)** Mice grouping and experiment timeline. **(B)** Escape latency during training days in Morris water maze test (MWT). ^***^ *P* < 0.001 *versus* the wild type (WT) control group. ^#^ *P* < 0.05 *versus* the APP/PS1 model group. **(C)** Time stayed in target quadrant in the probe test. **(D)** Numbers of target cross in the probe test. **(E)** Average swimming speeds. **(F)** Representative track images of mice during probe test in MWT. Data showed as mean ± SD. ^*^ *P* < 0.05, ^**^ *P* < 0.01,^***^ *P* < 0.001. n. s., no significant difference. WT, wild type; CTL, low dose of the total glycosides from *C. tubulosa*; CTM, medium dose of the total glycosides from *C. tubulosa*; CTH, high dose of the total glycosides from *C. tubulosa*.

The experimental design of the animal study was shown in Figure 2A. Morris water maze test was conducted to evaluate the effect of GCT on cognitive function. During the training days, the escape latency of mice in all groups showed a decreased trend with prolonged training (Figure 2B). Although the APP/PS1 mice exhibited significantly prolonged escape latencies when compared to the normal controls, high-dose GCT group showed shortened escape latency on day 5 when compared to the model group (*P* < 0.05, Figure 2B). In subsequent spatial probe test, mice in APP/PS1 model group spent significantly less time in the target quadrant (*P* < 0.001, Figure 2C) and showed fewer platform crossings (*P* < 0.01, Figure 2D) when compared to the normal controls. Notably, mice in medium- GCT group, high-dose GCT group, and donepezil group exhibited significantly increased target quadrant dwell time compared to the APP/PS1 model mice (*P* < 0.05), while mice in high-dose GCT group also demonstrating a marked increase in platform crossings (*P* < 0.05). No significant differences in average swimming speed were observed across groups, indicating that behavioral improvements were not attributable to changes in motor ability (Figure 2E). Post-platform removal, the normal control, donepezil, and high-dose GCT groups displayed trajectories biased toward the original platform quadrant, while the model, low-, and medium-dose GCT groups showed evenly distributed trajectories across all quadrants (Figure 2F). These data suggested that GCT, especially at the high dose (400 mg kg^-1^·d^-1^) in present study, improved the spatial learning and memory behavior of APP/PS1 mice. Since GCT at 400 mg kg^-1^·d^-1^ exhibited optimal therapeutic efficacy in APP/PS1 mice, our subsequent studies were conducted at this dose.

### 3.3 GCT reduced Aβ burden in APP/PS1 mice

**FIGURE 3:**
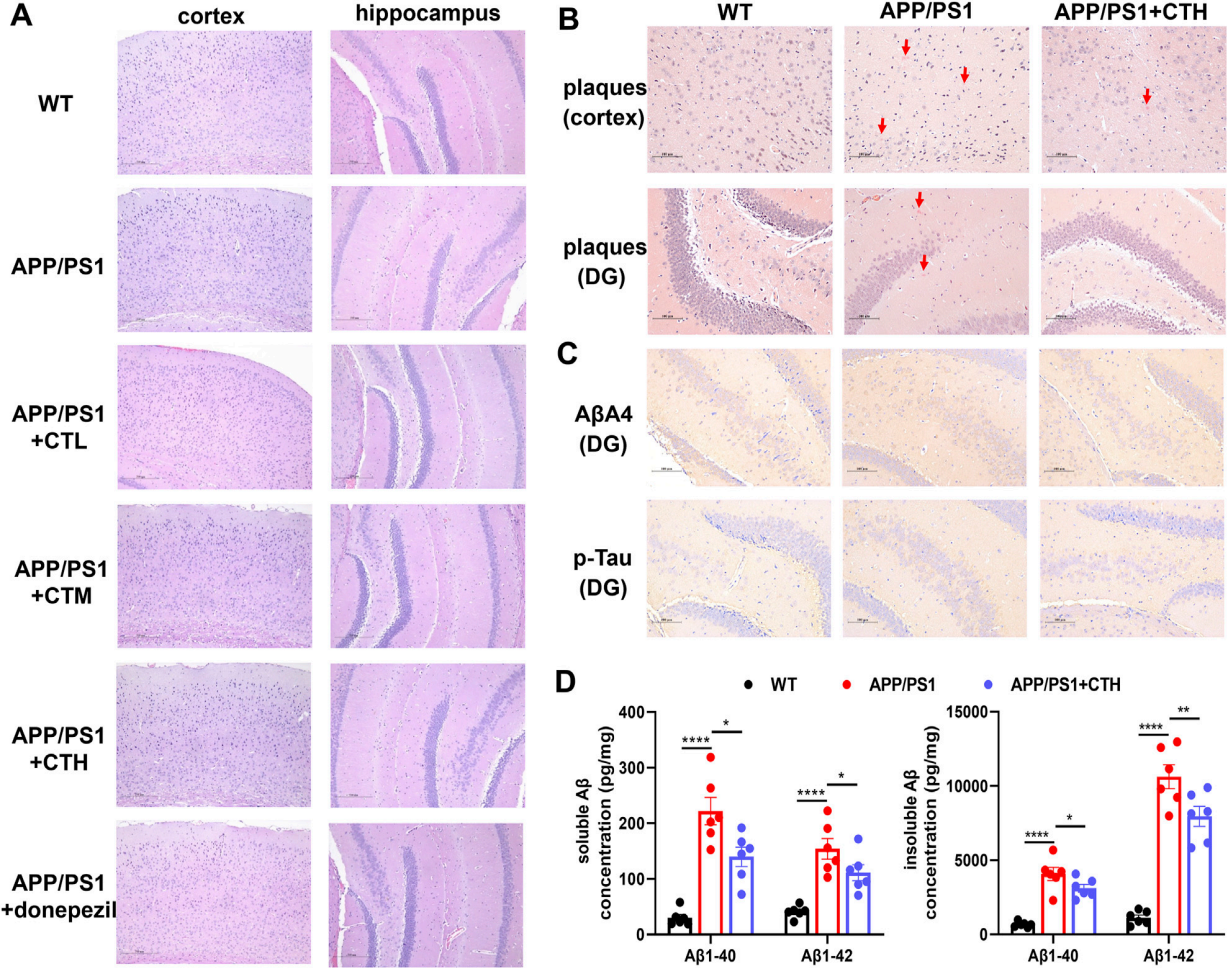
Effect of the total glycosides from *Cistanche tubulosa* on brain histopathology and biochemical markers. **(A)** H&E staining. **(B)** Congo red staining. **(C)** Immunohistochemistry for Aβ and p-Tau. **(D)** The contents of soluble and insoluble Aβ among different groups determined by ELISA assays. ^*^ *P* < 0.05, ^***^ *P* < 0.001. DG, dentate gyrus; WT, wild type; CTL, low dose of the total glycosides from *C. tubulosa*; CTM, medium dose of the total glycosides from *C. tubulosa*; CTH, high dose of the total glycosides from *C. tubulosa*.

H&E staining revealed normal cortical and hippocampal structures across all groups, with no edema, hemorrhage, or inflammatory infiltration (Figure 3A). Congo red staining showed no senile plaques in controls. The model group exhibited increased scattered senile plaques in the cortex and hippocampus, whereas the GCT group showed reduced cortical and hippocampal plaques (Figure 3B). Immunohistochemistry demonstrated weak p-Tau and Aβ expression in hippocampal neurons of controls. The model group exhibited an enhanced p-Tau and Aβ expression with increased positive cells, while the GCT group showed attenuated expression and fewer positive cells compared to the model group (Figure 3C). ELISA analyses further revealed significantly elevated soluble and insoluble Aβ1-40 and Aβ1-42 levels in the model group *versus* controls (*P* < 0.001). GCT treatment at 400 mg kg^-1^·d^-1^ significantly reduced both soluble and insoluble Aβ1-40 and Aβ1-42 levels in hippocampi of APP/PS1 mice (*P* < 0.05, Figure 3D).

### 3.4 GCT altered the gut microbial composition of APP/PS1 mice

**FIGURE 4:**
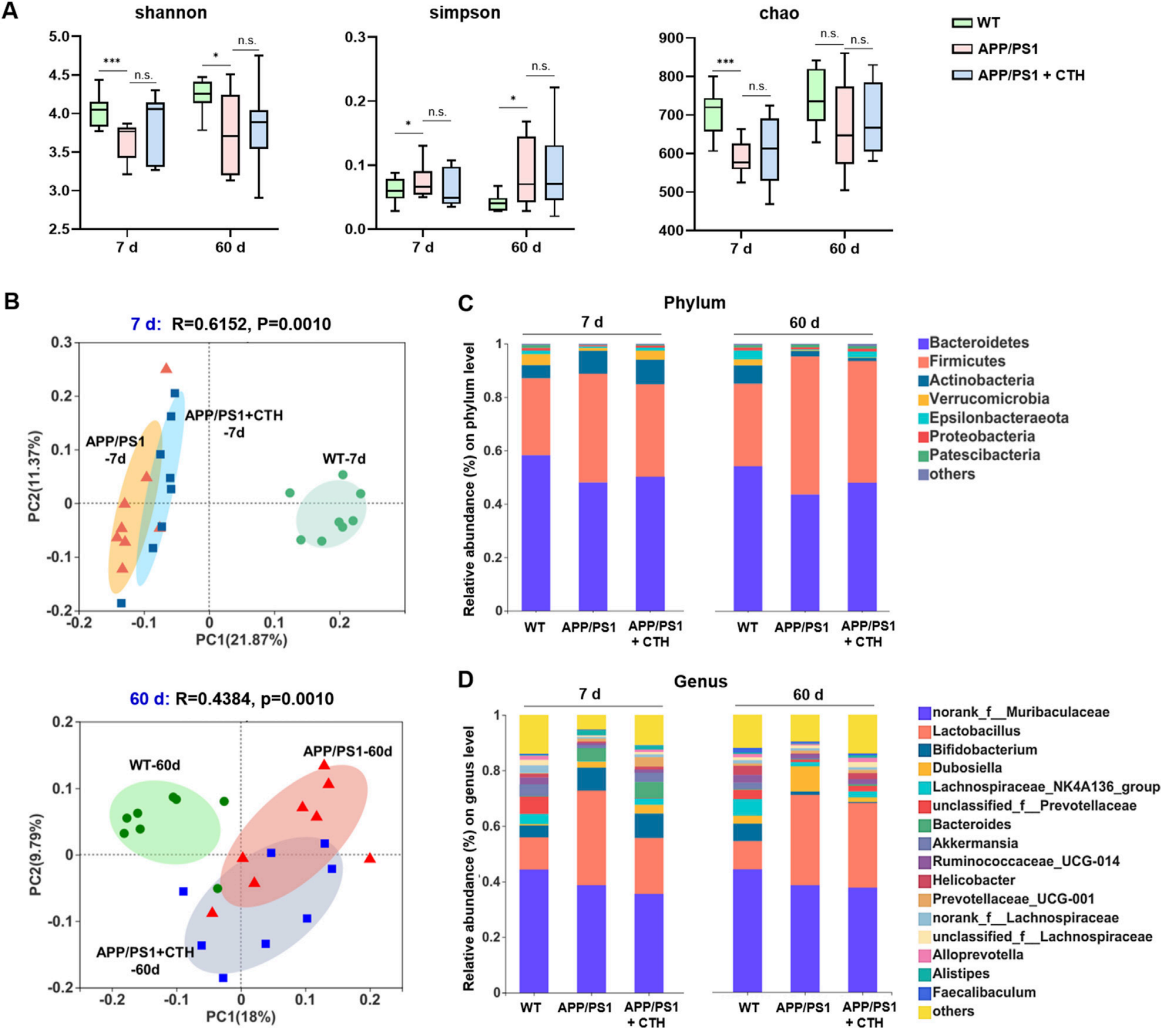
The effects of total glycosides from *Cistanche tubulosa* on the gut microbial diversity of APP/PS1 mice. **(A)** α-diversity at the 7th and 60th days of intervention, respectively: Shannon, Simpson, and Chao indexes. **(B)** β-diversity at the 7th and 60th days of intervention, respectively: plots from the principal coordinates analysis. **(C)** Community analysis based on phylum level. **(D)** Community analysis based on genus level. WT, wild type; CTH, high dose of the total glycosides from *C. tubulosa*. ^*^ *P* < 0.05; ^***^ *P* < 0.001; n. s., no significant difference.

Fecal samples were collected on day 7 and day 60 of GCT intervention and subjected to 16S rRNA sequencing. As shown in Figure 4A, the model group exhibited significantly reduced Shannon index and elevated Simpson indexes, as well as reduced Chao index of observed operational taxonomic units (OUTs) compared to the normal controls (*P* < 0.05), indicating diminished gut microbiota α-diversity and richness in APP/PS1 mice. Although high-dose GCT intervention failed to alter the alpha-diversity, the principal coordinates analysis (PCoA) based on weighted UniFrac distance demonstrated distinct microbial compositions among the control, APP/PS1 model, and high-dose GCT groups at both day 7 and day 60 (Figure 4B). When compared to the PCoA result at day 7 (Adonis: *R* ^2^ = 0.615, *P *= 0.001), more striking difference between the model group and GCT-treated group appeared at day 60 (Adonis: *R* ^2^ = 0.438, *P* = 0.001). Taxonomic composition analysis focused on the top-ranked taxa at two different levels (phylum and genus) further revealed the regulatory effect of GCT on gut microbiota composition of APP/PS1 mice. Consistent with the β-diversity results of PCoA, the gut microbiota composition of APP/PS1 mice showed significant changes within 7 days of GCT intervention (Figure 4C). These results suggested that short-term GCT intervention rapidly altered gut microbiota architecture, potentially preceding cognitive amelioration in APP/PS1 mice.

At the phylum level, *Firmicutes* and *Bacteroidetes* dominated the gut microbiota across all groups, but their ratios differed (Figure 4C). The ratio of *Firmicutes* to *Bacteroidetes* was 0.57 in controls, increased to 1.19 in APP/PS1 model group, and partially declined to 0.84 in GCT group after 60-day intervention. The relative abundance of *Epsilonbacteraeota* dropped in the model group (0.75% vs 3.32% in controls) and was restored to 2.35% after 60-day GCT treatment. Similarly, *Proteobacteria* abundance decreased in the model group (0.75% vs 1.07% in controls) but was elevated to 1.25% after GCT intervention (Figure 4C). At the genus level, GCT treatment also rebalanced the abundances of several main bacterial genera in APP/PS1 mice gut microbiota, such as *Akkermansia*, *Dubosiella*, *Lactobacillus*, and Lachnospiraceae_*NK4A136*_*group* since the 7th day of GCT intervention (Figure 4D).

**FIGURE 5:**
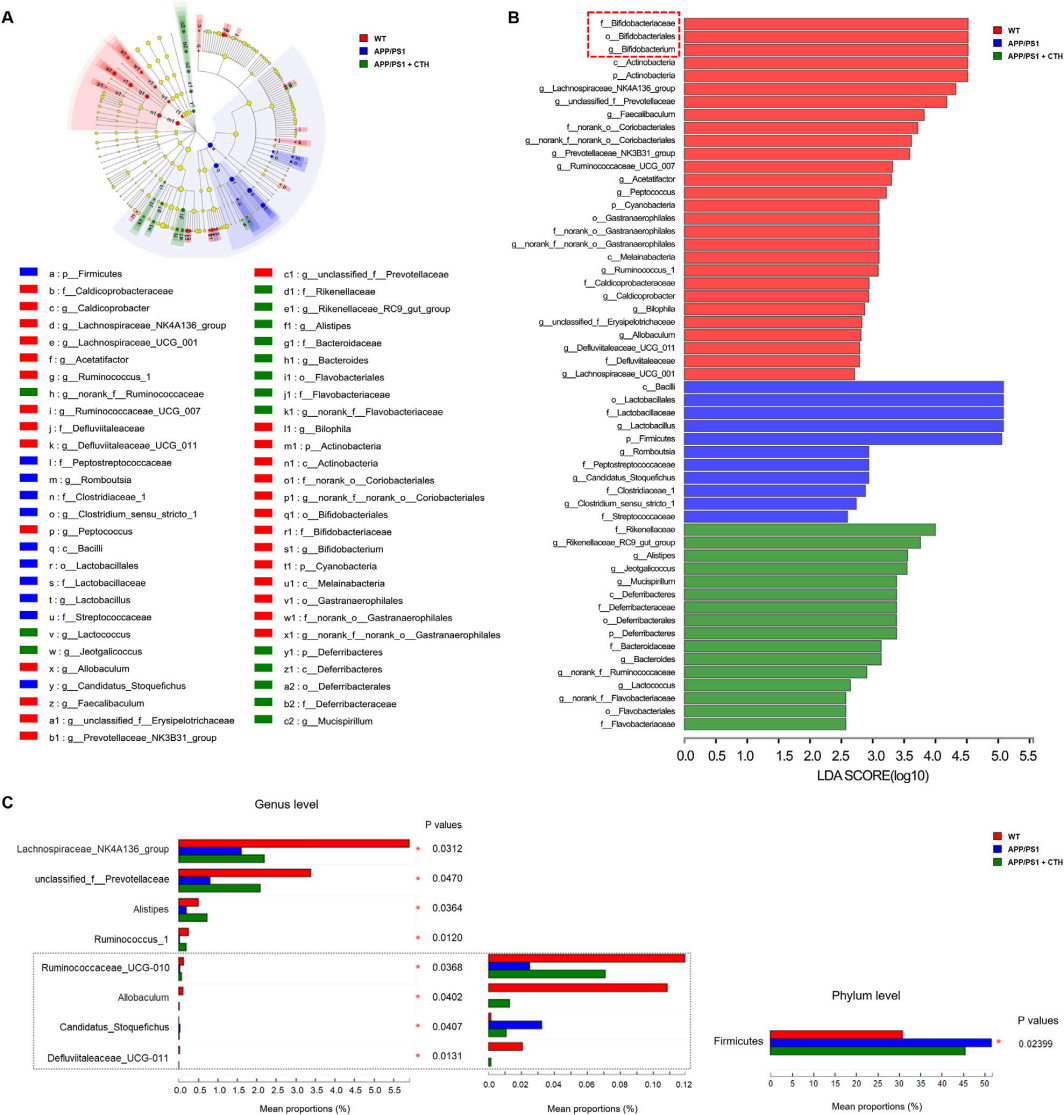
The total glycosides of *Cistanche tubulosa* reshaped the composition of gut microbiota in APP/PS1 mice at the 60th day of intervention. **(A)** Cladogram of different taxonomic compositions of the three groups by linear discriminant analysis effect size (LEfSe) (LDA score >2.0, *P* < 0.05). **(B)** The LDA scores (log 10) of differential bacterial taxa among the three groups provided by LEfSe. **(C)** The relative abundances of significantly altered gut microbiota at phylum and genus levels (*P* < 0.05). WT, wild type; CTH, high dose of the total glycosides from *C. tubulosa*. ^*^ *P* < 0.05.

To further elucidate the effect of GCT on the composition of gut microbiota, linear discriminant analysis effect size (LEfSe) method was employed to identify key microbiota with significant intergroup disparities at day 60, as this time point represented the study endpoint (LDA threshold = 2). The normal control group was characterized by dominant taxa such as *f_*Bifidobacteriaceae and *f_*Prevotellaceae. GCT intervention of 60 days mainly promoted the enrichment of beneficial bacteria from *f_*Rikenellaceae*, f_*Deferribacteraceae*,* and *f_*Bacteroidaceae (Figures 5A,B). Intergroup differences at day 60 were further analyzed using the Kruskal–Wallis rank sum test. Consistently, the GCT intervention also corrected the abnormal increase of *Firmicutes* at phylum level (Figure 5C). All above results indicated that the gut microbiota in APP/PS1 mice altered significantly, while GCT modulated the composition of gut microbiota.

### 3.5 GCT altered the metabolism of fatty acids in serum of APP/PS1 mice

**FIGURE 6:**
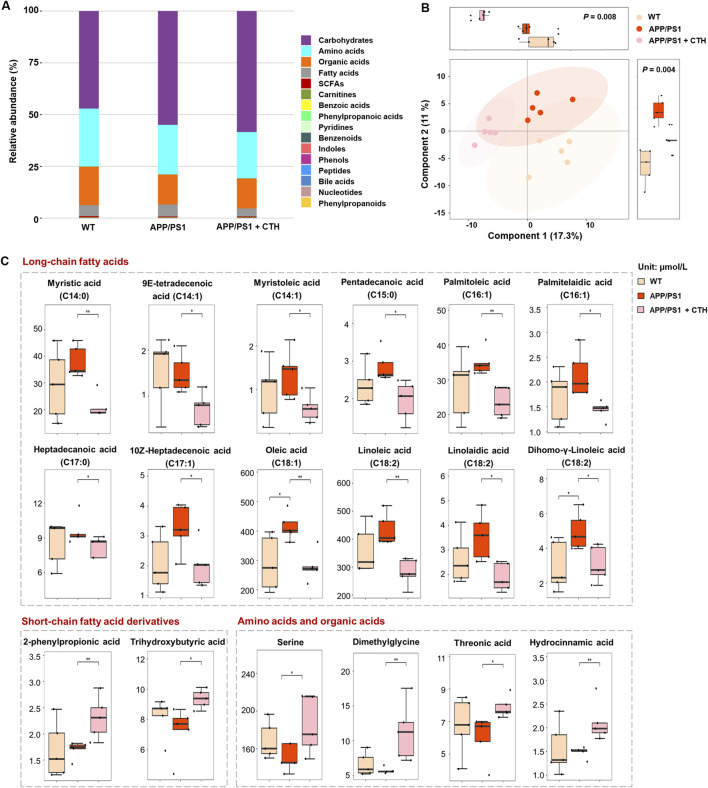
The effect of total glycosides of *Cistanche tubulosa* (GCT) on serum metabolic profile of APP/PS1 mice. **(A)** The composition of serum metabolome in wild type (WT) mice, APP/PS1 mice, and GCT-treated APP/PS1 mice. **(B)** The score plot of partial least squares-discriminant analysis. **(C)** The content of metabolites significantly altered in serum after GCT treatment. CTH, high dose GCT. ^*^ *P* < 0.05, ^**^ *P* < 0.01.

Microbiota-derived metabolites participate in host metabolism through multiple pathways. Investigating host metabolic phenotypes could provide a comprehensive understanding of GCT’s metabolic regulatory effects and its anti-dementia mechanisms through the “gut-brain” axis. Consequently, a targeted metabolomics analysis was performed on mice serum and cerebral tissue, enabling the simultaneous quantification of 306 metabolites, including 201 gut microbiota-associated metabolites. A total of 209 metabolites were successfully detected and quantified in mice serum from control, APP/PS1, and APP/PS1 + high-dose GCT group, including carbohydrates (54.26%, content ratio), amino acids (24.68%), organic acids (15.4%), fatty acids (4.79%), and others (0.87%) (Figure 6A). The observed reduction from the predefined panel of 306 target metabolites to the final quantifiable 209 ones is due to metabolites either falling below the limit of detection or being absent in mice serum, as expected when applying a fixed commercial panel.

Partial least squares-discriminant analysis (PLS-DA) revealed distinct metabolic profiles among the three groups, with pronounced differences between the normal control and APP/PS1 model groups, while the high-dose GCT group exhibited an intermediate profile (Figure 6B). Differential metabolites among the groups were identified based on univariate statistical analysis. A total of 18 metabolites showed statistically significant alterations in serum of GCT-treated group compared with those in APP/PS1 model group. Among these, 12 long chain fatty acids with unsaturation degree of 0–2 showed significant decrease in circulating levels after 60-day GCT treatment (Figure 6C). Notably, the serum levels of two short-chain fatty acid derivatives, 2-phenylpropionic acid and trihydroxybutyric acid, were significantly elevated following GCT intervention (*P* < 0.05). Furthermore, the precursors of 2-phenylpropionic acid, serine and dimethylglycine, also exhibited significant upregulation post-GCT treatment (*P* < 0.05).

### 3.6 GCT corrected the metabolic disruptions of fatty acids in brain of APP/PS1 mice

**FIGURE 7:**
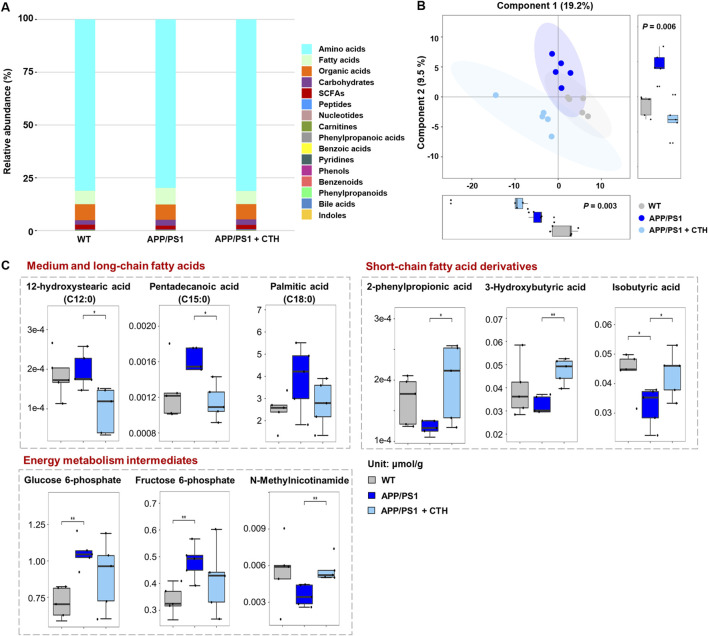
The effect of total glycosides of *Cistanche tubulosa* (GCT) on metabolic profile of cerebral tissue from APP/PS1 mice. **(A)** The composition of brain metabolome in wild type (WT) mice, APP/PS1 mice, and GCT-treated APP/PS1 mice. **(B)** The score plot of partial least squares-discriminant analysis. **(C)** The content of metabolites significantly altered in cerebral tissue after GCT treatment. CTH, high dose GCT. ^*^ *P* < 0.05, ^**^ *P* < 0.01.

In cerebral samples of mice, a total of 194 metabolites were determined by the targeted metabolomics approach. Distinct from the serum metabolic profile, amino acids accounted for 80.75% of detected metabolites in cerebral tissue across the three experimental groups, followed by organic acids (7.38%) and fatty acids (6.82%) as the other predominant categories (Figure 7A). Subsequent PLS-DA analysis revealed intergroup metabolic disparities (Figure 7B). Univariate statistical comparisons further revealed metabolites that demonstrated significant reversal in cerebral tissue following GCT intervention. As shown in Figure 7C, the contents of three saturated fatty acids, including 12-hydroxystearic acid (C12:0), pentadecanoic acid (C15:0), and palmitic acid (C18:0), were elevated in APP/PS1 mice and significantly attenuated following GCT intervention. Conversely, three short-chain fatty acids, including 3-hydroxybutyric acid, isobutyric acid, and 2-phenylpropionic acid, exhibited significant reduction in APP/PS1 model mice, with their levels restored post-GCT intervention. In addition, the aberrant changes in contents of glucose 6-phosphate, fructose 6-phosphate, and N-methylnicotinamide, three intermediates involved in energy metabolism, were also attenuated by GCT.

These results were well collaborated with those metabolic changes observed in circulation. Specifically, the contents of short-chain fatty acids, such as 2-phenylpropionic acid and butyric acid derivatives, significantly increased after GCT intervention in both serum and cerebral tissue, with 3-hydroxybutyric acid demonstrating promising neuroprotective efficacy against dementia pathogenesis (Kachoueiyan et al., 2025; Madhavan et al., 2025; Ge et al., 2023). Moreover, the results revealed pathological accumulation of several saturated long-chain fatty acids (LCFAs) in circulatory systems and cerebral parenchyma of APP/PS1 mice, with GCT intervention achieving markedly normalization of these pro-inflammatory metabolites. The gut microbiota-dependent nature of fatty acid metabolism suggests that the GCT-induced gut microbiota remodeling potentially contributes to its observed anti-dementia effect through metabolic reprogramming, indicating modulation of fatty acids-homeostasis as a key mechanistic basis for GCT’s therapeutic effect.

### 3.7 GCT decreased the neuroinflammation of APP/PS1 mice

**FIGURE 8:**
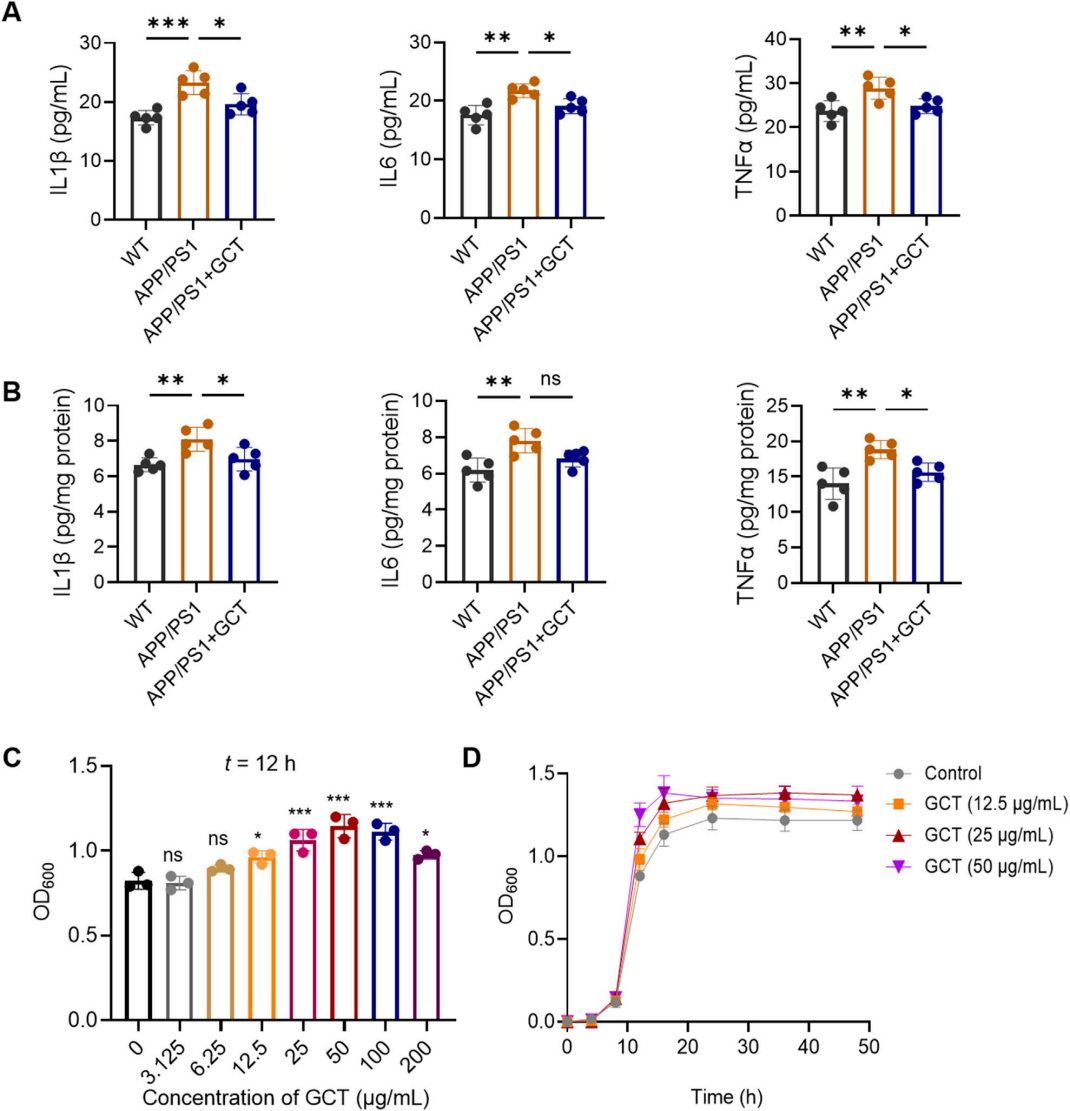
The total glycosides of *Cistanche tubulosa* (GCT) decreases the proinflammatory factors *in vivo* and promotes the growth of *Akkermansia muciniphila in vitro*. **(A)** Levels of proinflammatory factors in mice serum. **(B)** Levels of proinflammatory factors in mice cerebral tissue. **(C)** Effect of GCT on the growth of *A. muciniphila*. **(D)** The growth curves of *A. muciniphila*. ^*^ *P* < 0.05, ^**^ *P* < 0.01, ^***^ *P* < 0.001; ns, no significance.

SCFAs, such as propionate and butyrate, have been reported to exert anti-inflammatory effects, whereas both saturated and monounsaturated LCFAs exhibit pro-inflammatory properties. Strikingly, GCT intervention significantly increased these anti-inflammatory SCFAs while concurrently reducing pro-inflammatory LCFAs (Figures 6C, 7C). For neuroinflammation plays a pivotal role in the pathogenesis of AD (Leng and Edison, 2021), then we measured the inflammatory markers from different groups. As illustrated in Figure 8, the levels of IL-1β, IL-6, and TNF-α in both serum and brain of APP/PS1 mice were markedly upregulated compared to the normal controls, indicating aberrantly elevated inflammation in both circulation and brain tissue (*P* < 0.01). In contrast, these pro-inflammatory cytokines were significantly downregulated in the GCT-treated group compared to the APP/PS1 model group (*P* < 0.05). These data demonstrate that GCT effectively attenuates neuroinflammation in AD mice, which could be mediated by the altered and their mediated fatty acids.

### 3.8 GCT promoted the proliferation of *Akkermansia muciniphila in vitro*

**FIGURE 9:**
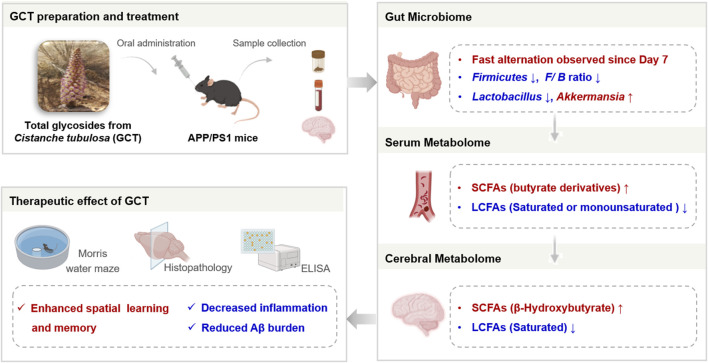
The total glycosides of *Cistanche tubulosa* (GCT) ameliorate cognitive decline in APP/PS1 mice probably through modulation of gut microbiota and fatty acid metabolism.

*Akkermansia muciniphila*, a widely recognized probiotic in the gut microbiota, is known to promote the production of SCFAs including propionate and butyrate. *Akkermansia muciniphila* itself as well as its metabolites have been proven to alleviate dementia-like symptoms in APP/PS1 mice (Ou et al., 2020) and 5×FAD mice (Wang et al., 2025). Although the 16S rRNA sequencing has revealed a significantly enhanced level of *A. muciniphila* after 7 days’ GCT intervention *in vivo* (Figure 4D), the effect of GCT on *A. muciniphila* still needs validation to further elucidate the mechanisms of GCT in treating AD. Our subsequent *in-vitro* study found that GCT significantly promoted the growth of *A. muciniphila* pure-cultured strain at 12 h in a dose-dependent manner (Figure 9A). Further growth curve analysis indicated that the logarithmic period for *A. muciniphila* was observed to end at 16 h, while GCT at concentration of 12.5, 25, and 50 μg/mL all enhanced the proliferation of *A. muciniphila* (Figure 9B).

## 4 Discussion

Globally, over 50 million people lived with dementia in 2020, with prevalence projected to double every 2 decades, reaching 82 million by 2030 and 152 million by 2050 (GBD, 2019 Dementia Forecasting Collaborators, 2022). In China, the socioeconomic burden of dementia is anticipated to exceed 3.2 trillion yuan by 2030 (Zhu et al., 2023). AD, accounting for 60%–80% of dementia cases, remains a therapeutic challenge due to limited preventive or curative interventions. Medicinal and dietary herb have demonstrated advantages in managing chronic and age-related diseases, highlighting the untapped potential of natural products and herbal formulations for anti-AD drug development. PhGs from *Cistanche* herbs exhibit well-documented anti-dementia effects, yet their low oral bioavailability and mechanistic underpinnings remain incompletely understood. The mechanism through which GCT displayed the anti-AD effects remains unclear.

The present study demonstrates that GCT effectively ameliorate cognitive deficits and pathological hallmarks in APP/PS1 double-transgenic mice. GCT intervention significantly improved spatial learning and memory as well as reduced Aβ deposition. Furthermore, multi-omics analyses firstly revealed that GCT reshaped gut microbiota composition and rectified systemic and cerebral metabolic disturbances in AD-model mice, particularly in fatty acid and energy metabolism pathways. The *in-vitro* experiment further demonstrated that *A. muciniphila* might have played the crucial role in aforementioned changes caused by GCT. These findings not only elucidate the apparent paradox between the low bioavailability and potent neuroprotective effects of phenylethanoid glycosides, but also indicate that GCT serves as a promising therapeutic candidate for AD, acting via gut microbiota modulation and metabolic reprogramming.

APP/PS1 mice were selected for its accelerated Aβ accumulation and AD-associated memory deficits, which begins at 6 months of age and progressively worsens, mimicking key features of human AD (Cao et al., 2021). By initiating treatment at 6 months and last for 2 months, this study targeted a stage of established amyloidosis and cognitive decline, allowing evaluation of GCT’s therapeutic potential in reversing advanced AD-like pathology. The observed improvements in Aβ burden and cognitive performance validate the model’s relevance for testing anti-AD interventions. Consistent with previous reports (Chen et al., 2024), APP/PS1 mice exhibited reduced gut microbial diversity and altered taxonomic composition, characterized by decreased beneficial taxa (e.g., *Bacteroidetes*) and increased pro-inflammatory genera (e.g., Lactobacillaceae, *Dubosiella*). GCT intervention restored microbial balance, enriching Rikenellaceae and Bacteroidaceae, families associated with anti-inflammatory and neuroprotective effects. Notably, in the normal gut microbiota, *Firmicutes* and *Bacteroidetes* are two significant phyla, while the increased *Firmicutes*-to-*Bacteroidetes* ratio (F/B) has been associated with multiple pathological states (Lee et al., 2021; Strati et al., 2017). In this study, an increased *F*/*B* was also observed in APP/PS1 mice, which was decreased after GCT intervention. It has been revealed that *Firmicutes* and *Bacteroidetes* are important for intestinal energy metabolism, yet an increase in F/B caused metabolic disruptions (He et al., 2018; Sweeney and Morton, 2013).

*Akkermansia muciniphila* has been shown to have a significant impact on inflammation and neurodegeneration. In AD patients, there is a decrease in the abundance of *A. muciniphila* and a decrease in propionic acid in both fecal and blood samples (Wang et al., 2025). Probiotic interventions targeting *A. muciniphila* show promise in the AD treatment. Treatment with *A. muciniphila* reduced regulated cognitive impairment and hippocampal NLRP3-mediated neuroinflammation through improving intestinal barrier function and attenuating Th17 responses in the gut, central nervous system, and lymphoid tissues of mice (Li et al., 2025). A cocktail of unique probiotics, including strains of *A. muciniphila*, significantly reduced cognitive decline and AD pathology markers in a humanized AD mouse model. The beneficial effects were linked to a reduced inflammation in both systemic circulation and the brain (Prajapati et al., 2025). In another study, *A. muciniphila* protected against dopamine neurotoxicity in a Parkinson’s disease model by modulating butyrate to inhibit microglia-mediated neuroinflammation, suggesting its potential in treating neurodegenerative diseases (Xu et al., 2025). These findings directly support the mechanistic relevance of GCT-induced A. muciniphila enrichment observed in our study, strengthening the biological rationale for its potential benefits in AD.

In recent years, metabolomics has been increasingly applied to investigate gut microbiota metabolism and host-microbiota co-metabolism. Metabolomics enables rapid profiling of gut microbiota-associated metabolites, reflecting the metabolic landscape of microbial activity. Additionally, microbiota-derived metabolites participate in host metabolism through multiple pathways. Investigating host metabolic phenotypes may comprehensively reveal GCT’s metabolic regulatory effects and anti-dementia mechanisms through the “gut-brain” axis. In this study, targeted metabolomics further revealed that GCT normalized dysregulated metabolites linked to microbial activity, including SCFAs and LCFAs. This integrative approach underscores the gut-brain axis as a critical mediator of GCT’s anti-AD effects, bridging microbial compositional changes to functional metabolic outcomes.

Aberrant fatty acid metabolism is a hallmark of AD. Elevated serum levels of oleic acid, linolenic acid, and linoelaidic acid in APP/PS1 mice align with clinical reports linking these metabolites to dementia risk (Honda et al., 2019). GCT significantly reduced these pro-inflammatory fatty acids, suggesting a mechanism involving peripheral immune modulation. Furthermore, several saturated LCFAs (such as oleic acid and palmitic acid), as well as monounsaturated LCFAs (such as linoleic acid and Palmitoleic acid), known to promote Aβ and tau aggregation (Mill and Li, 2022; Qian et al., 2023), were also normalized post-GCT treatment. These findings highlight GCT’s role in mitigating lipid-driven neuroinflammation, a key contributor to AD progression.

Butyrate is a major SCFA constituting over 60% of gut microbiota-derived SCFAs through bacterial fiber fermentation. In this study, intervention with GCT significantly elevated cerebral levels of 3-hydroxybutyric acid (β-hydroxybutyrate) and isobutyric acid in AD model mice. Notably, GCT restored brain levels of neuroprotective SCFAs, including 3-hydroxybutyric acid and isobutyric acid. These metabolites enhance secretion of brain derived neurotrophic factors and counteract neuronal degeneration (Bairamian et al., 2022). In patients with AD and its animal models, decreased levels of β-hydroxybutyrate have been implicated in disease pathological progression (Kachoueiyan et al., 2025; Madhavan et al., 2025). Studies suggest that it plays a critical role in AD pathogenesis. Exogenous β-hydroxybutyrate supplementation reduced plaque formation, microgliosis, and caspase-1 activation in the 5×FAD AD mouse (Shippy et al., 2020). Furthermore, β-hydroxybutyrate ameliorates AD pathology by modulating β-hydroxybutyrylation (Kbhb), a post-translational modification of tricarboxylic acid (TCA) cycle-related enzymes. In APP/PS1 AD model mice, Kbhb modifications of TCA enzymes were significantly reduced during pathological stages. However, β-hydroxybutyrate supplementation markedly restored Kbhb modification levels and enzymatic activity, thereby enhancing ATP production and mitigating amyloid-β plaque pathology (Han et al., 2025). Disrupted cerebral glucose metabolism and Warburg-like glycolytic shifts have also been characterized in AD (Chen and Zhong, 2013; Dewanjee et al., 2022). Elevated glucose 6-phosphate and fructose 6-phosphate in APP/PS1 mice reflect impaired energy homeostasis, which GCT effectively reversed.

Collectively, GCT ameliorates AD pathology by modulating gut microbiota composition and associated metabolic networks, particularly fatty acid and energy pathways. Despite the low oral bioavailability of PhGs, their efficacy likely arises from microbial metabolic cooperation, underscoring the gut-brain axis as a promising target for dietary interventions supporting brain health. Furthermore, the dried succulent stems of both *C. tubulosa* and *C. deserticola* are traditionally consumed as nourishing foods in China and across various Asian regions. Within this context, *C. tubulosa* was selected for this study due to its reported superior bioactivity in supporting neurological health. Comparative studies highlight that *C. tubulosa* extracts exhibit stronger effects on mood regulation than *C. deserticola* (Fan et al., 2022). Moreover, PhGs, the key bioactive components associated with cognitive support, are more enriched in *C. tubulosa* (Liu et al., 2019). To maximize PhG yield, 75% ethanol reflux extraction was employed, yielding the GCT extract rich in these glycosides. The mediating role of gut microbiota proves particularly crucial in explaining the therapeutic effects of many TCM components with low oral bioavailability. Investigating the regulatory effects on gut microbiota composition and microbial metabolite production could provide novel perspectives for elucidating their pharmacological mechanisms.

In addition, GCT contains diverse bioactive glycosides that likely exert therapeutic effects through multiple targets and signaling pathways. Our comprehensive analysis reveals that GCT induces multidimensional modulatory effects on gut microbiota architecture. Taxonomic profiling demonstrates significant restoration of the *Firmicutes*-to-*Bacteroidetes* ratio alongside enrichment of beneficial genera including *Akkermansia* and *Dubosiella*, indicating enhanced microbial ecosystem resilience. Functional metabolomics further indicates GCT-mediated upregulation of SCFA biosynthesis coupled with downregulation of saturated LCFA production. These coordinated shifts suggest GCT remodels microbial communities toward a health-associated, anti-inflammatory phenotype, potentially disrupting the gut dysbiosis-inflammation axis implicated in AD pathogenesis. Critically, this represents an integrated regulatory action that not attributable to isolated bacterial strains or singular metabolic pathways, but rather the synergistic effects of multiple components. Given the multifactorial pathogenesis of AD, involving concurrently dysregulated neuroinflammatory cascades, proteostasis failure, and metabolic dysfunction, this systems-level therapeutic approach may offer superior efficacy in modulating disease progression compared to single-metabolite or single-strain interventions. Furthermore, the established safety profile of Cistanche Herba, recognized for centuries as a dual-purpose medicinal and edible plant within traditional pharmacopeias, provides a robust foundation for clinical translation. Collectively, these findings position GCT as a promising complementary therapeutic candidate worthy of further development.

## 5 Conclusion

In this study, we provide the first evidence that GCT alleviates cognitive deficits and Aβ pathology in APP/PS1 mice primarily through remodeling the gut microbiota and rectifying dysregulated fatty acid metabolism, with *Akkermansia* playing a significant role. This study not only clarified the mechanism by which Cistanches Herba alleviate cognitive decline from a novel perspective, but also added new evidences for its traditional use as anti-dementia therapy.

## Data Availability

The original contributions presented in the study are included in the article/Supplementary Material, further inquiries can be directed to the corresponding author.
