# Supplementary material for: Cistanche tubulosa glycosides ameliorate cognitive decline in APP/PS1 mice via modulation of gut microbiota and fatty acid metabolism: insights from multi-omics and experimental validation
Source: Front Pharmacol. 2025 Aug 8;16:1662336. doi: 10.3389/fphar.2025.1662336 (PMC12370757; doi:10.3389/fphar.2025.1662336)
Supplement: Supplementary file 1 [file Supplementaryfile1.docx]

Supplementary Material

***Cistanche tubulosa* glycosides ameliorate** **cognitive decline in APP/PS1 mice via modulation of gut microbiota and fatty acid metabolism: Insights from multi-omics and experimental validation**

**Rui Hou ^1†^, Wei Song ^2†*^, Yi Nan ^3^, Yiyi Gong ^2^, Jieying Liu ^2^, Jialin Liu ^2^**

^1^Department of General Surgery, Peking Union Medical College Hospital, Chinese Academy of Medical Sciences & Peking Union Medical College, Beijing, 100730, China

^2^Center for Biomarker Discovery and Validation, National Infrastructures for Translational Medicine, Institute of Clinical Medicine, Peking Union Medical College Hospital, Chinese Academy of Medical Science & Peking Union Medical College, Beijing, 100730, China

^3^Graduate School, Tianjin University of Traditional Chinese Medicine, Tianjin, 301617, China

†These authors contributed equally to this work and share first authorship

*** Correspondence:**Wei Song
[sw-yy1990@163.com](mailto:sw-yy1990@163.com)

**Sample Preparation for Targeted Metabolomics Analysis**

**1. Serum samples**

Samples were thawed on ice-bath to diminish sample degradation. 25uL of serum was added to a 96-well plate. Then the plate was transferred to a Biomek4000 workstation (Biomek 4000, Beckman Coulter, Inc., Brea, California, USA).120 μL ice cold methanol with partial internal standards was automatically added toeach sample and vortexed vigorously for 5 min. The plate was centrifuged at 4000g for 30 min (Allegra X-15R, Beckman Coulter, Inc., Indianapolis, IN, USA). Then the plate was returned to the workstation. 30 uL of supernatant was transferred to a clean 96-well plate, and 20 μL of freshly prepared derivative reagents was added to each well. The plate was sealed and the derivatization was carried out at 30°C for 60 min. After derivatization, 330 μL of ice-cold 50% methanol was added to dilute the sample. Then the plate was stored at -20°C for 20 minutes and followed by 4000 g centrifugation at 4 °C for 30 min. 135 μL of supernatant was transferred to a new 96-well plate with 10 μL internal standards in each well. Serial dilutions of derivatized stock standards were added to the left wells. Finally, the plate was sealed for LC-MS analysis.

**2. Tissue samples**

10 mg brain tissue sample was mixed with 10 pre-chilled zirconium oxide beads and 20 μL of deionized water in microcentrifuge tube. The sample was homogenated for 3 min and 120 μL of Methanol containing internal standard was added to extract the metabolites. The sample was homogenated for another 3 min and then centrifuged at 18000 g for 20 min. Then the supernatant was transferred to a 96-well plate. The following procedures were performed on the Biomek 4000 workstation. 20 μL of freshly prepared derivative reagent was added to each well. The plate was sealed and the derivatization was carried out at 30°C for 60 min. After derivatization, the sample was evaporated for 2 h. 330μL of ice-cold 50% methanol solution was added to reconstitute the sample. Then the plate was stored at -20°C for 20 min and followed by 4000 g centrifugation at 4 °C for 30 min. 135 μL of supernatant was transferred to a new 96-well plate with 10 μL internal standards in each well. Serial dilutions of derivatized stock standards were added to the left wells. Then the plate was sealed for LC-MS analysis.
